# An 8-week ketogenic diet improves exercise endurance and liver antioxidant capacity after weight loss in obese mice

**DOI:** 10.3389/fnut.2023.1322936

**Published:** 2023-12-29

**Authors:** Ying Wang, Yunlong Dong, Ying Zhang, Jiabao Yan, Cuiru Ren, Hong Ma, Zhenwei Cui

**Affiliations:** ^1^Centre for Sport Nutrition and Health, Centre for Nutritional Ecology, School of Physical Education (Main Campus), Zhengzhou University, Zhengzhou, China; ^2^Sports Department, Xi’an International Studies University, Xi’an, China

**Keywords:** ketogenic diet, obesity, exercise performance, oxidative stress, oxidation resistance

## Abstract

Evolving evidence supports the role of the ketogenic diet (KD) in weight loss. However, no coherent conclusions are drawn on its impact on the effect of KD on exercise and antioxidant capacity after weight loss in obese individuals. We evaluated the exercise performance, energy metabolism and antioxidant capacity of mice after weight loss using high-fat diet-induced obese mice, and used KD and normal diet (ND) intervention, respectively, to provide a theoretical basis for further study of the health effects of KD. Our results showed that the 8-week KD significantly reduced the body weight of obese mice and improved the performance of treadmill exercise, but had no significant effect on grip strength. Serum biochemical results suggest that KD has the risk of elevating blood lipid. In liver tissue, KD significantly reduced the level of oxidative stress and increased the antioxidant capacity of the liver. Our findings suggest that the intervention with KD led to weight loss, modulate energy metabolism and improve aerobic exercise endurance in obese mice. Despite its antioxidant potential in the liver, the utilization of KD still requires caution. This study underscores the need for further investigation into the health impacts of KD, especially in regard to its potential risks.

## 1 Introduction

The latest study by the World Health Organization in 2022 shows that the number of deaths worldwide caused by overweight and obesity is currently over 1.3 million people each year, with this number increasing annually. This escalation in cases has elevated obesity to a significant global health problem, posing a threat to individuals around the world. Obesity not only gives rise to a range of metabolic diseases, such as type 2 diabetes, hypertension, and non-alcoholic fatty liver disease. Furthermore, it speaks an increase in the secretion of inflammatory factors within the body. Chronic obesity adversely affects the body’s antioxidant capacity and exercise performance (1, 2). To combat this issue, various interventions have been suggested, including exercise, nutrition, and drug therapy. Of these interventions, exercise and nutrition have gained widespread recognition as effective means of combating obesity. Increasing physical activity levels to enhance energy expenditure, along with reducing calorie intake through dietary modifications, are common approaches adopted for weight loss. Unhealthy fat consumption plays a crucial role in the development of food-induced obesity (3). However, the ketogenic diet, a dietary intervention characterized by high fat and low carbohydrate intake, has shown promise in addressing obesity (4). Obesity’s reduction in mobility is strongly linked to the chronic inflammation it causes, as well as skeletal muscle atrophy and decreased cardiopulmonary function (5, 6).

The ketogenic diet was initially proposed in the 2020s as a treatment for epilepsy (7). This diet strictly limits carbohydrate intake, has a moderate protein intake and is high in fat (8). It works by simulating the body’s starvation state, prompting the body to produce ketone bodies for energy and promoting weight loss (9). In addition, the ketogenic diet improves the body’s antioxidant capacity and reduces levels of oxidative stress. For instance, Ma et al. found that a ketogenic diet reduced levels of oxidative stress markers ALT and BUN in mice (10). Similarly, research demonstrated that a short-term ketogenic diet significantly increased antioxidant capacity and glutathione peroxidase activity in rat liver (11). Furthermore, Huang et al. observed that an 8-week ketogenic diet not only reduced the production of oxidative stress markers but also increased aerobic capacity in exhausted mice (12). However, contrasting results have been reported by Wesley et al., who reported that a prolonged ketogenic diet decreased skeletal muscle glutathione levels and increased reactive oxygen species (ROS) (13), thereby negatively impacting mitochondria in skeletal muscle. Recently, the potential effects of dietary antioxidants have been the subject of many scientific studies on their role in cancer prevention (14–16). A previous meta-analysis showed that higher dietary total antioxidant capacity (TAC) intake is associated with a reduced risk of different types of cancer (17). And higher dietary TAC was related to reduced prevalence of central obesity, reduced waist circumference and triglyceride concentrations (18). Notably, a ketogenic diet was also found to increase the volume of mitochondria in liver and skeletal muscle, but not their antioxidant capacity (19). It is worth considering that ketogenic diets, being extreme diets with a very high fat content, may negatively affect blood lipids, leading to elevated triglycerides and cholesterol, which increase the incidence of cerebral infarction and heart disease (20).

At present, there are numerous successful cases of weight loss achieved through the implementation of a ketogenic diet. Studies on the improvement of antioxidant capacity through such a diet are also not uncommon. However, there is a scarcity of research on the impact of antioxidant capacity and exercise performance following weight loss in obese individuals. In addition, most of the existing studies on the effects of ketogenic diet on exercise performance have primarily focused on individuals of normal weight. Contrarily, research based on obese individuals is limited and inconsistent. For instance, Huang et al. found that a ketogenic diet improved treadmill endurance in mice (12). However, population-based studies have shown that ketogenic diets did not enhance exercise capacity in women participants (21). In addition, the ketogenic diet also has adverse effects on the body, such as increased fat, decreased cardiorespiratory capacity, and increased triglycerides (19, 22). Hence, it is essential to delve deeper into whether a ketogenic diet can improve antioxidant capacity and exercise performance, or if it may cause further damage to the body when used as a means of fat loss. In view of this, the present study aims to investigate the effects of a ketogenic diet on body weight, energy metabolism, blood lipids, antioxidant capacity and exercise performance in obese mice induced by a high-fat diet. Ultimately, this research will provide valuable insight into the potential use of a ketogenic diet in obese individuals.

## 2 Materials and methods

### 2.1 Animals and diets

**Figure 1:**
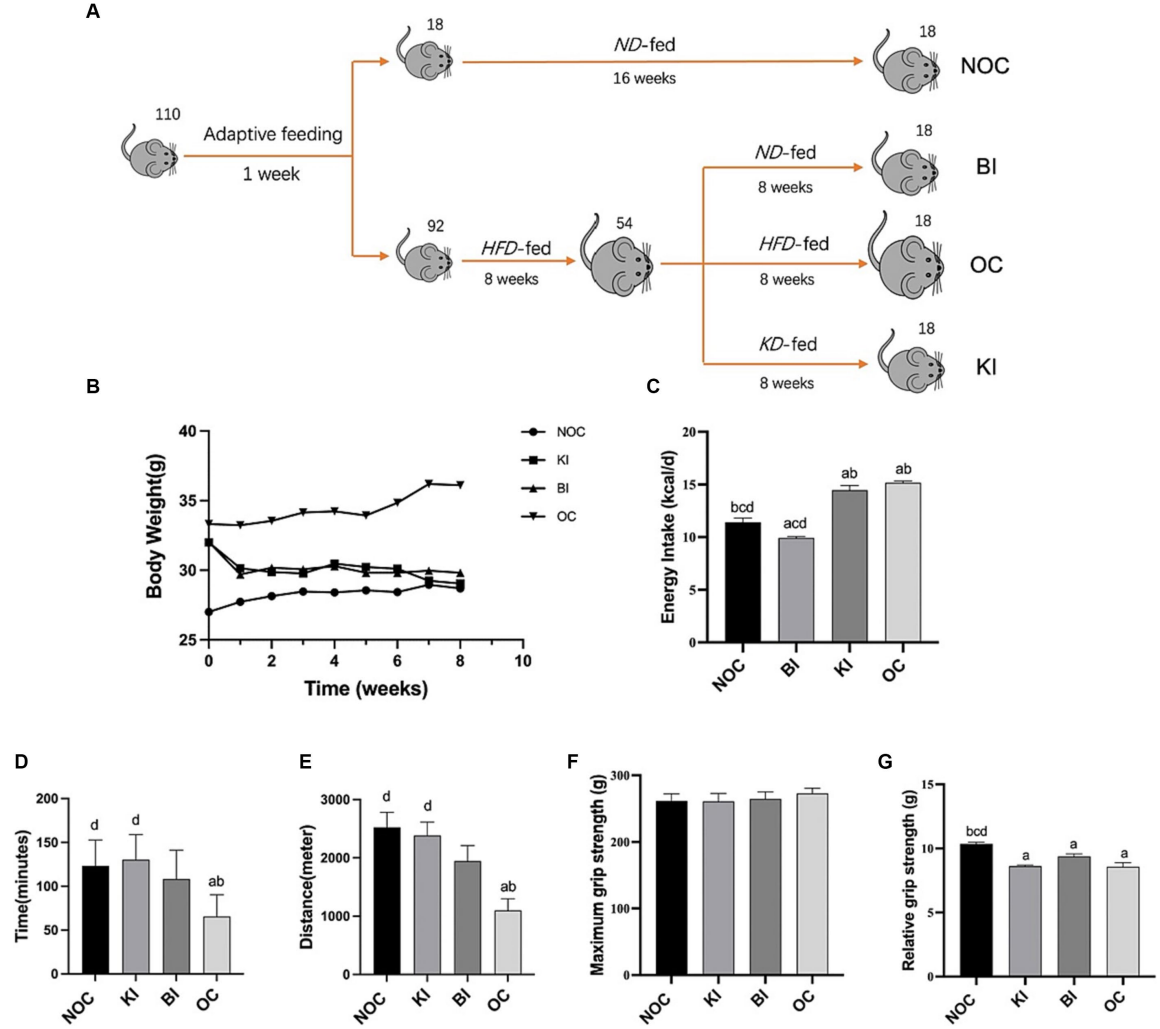
Dietary intervention design of model mice. **(A)** Dietary intervention design and grouping graph of obese mice during the 8-week intervention period. **(B)** Changes in body weight of the 4 groups of mice during the intervention (*n* = 6). **(C)** Average energy intake (kcal/d) of each group at week 8 (*n* = 6). **(D)** Time to one-time exhaustion in each group of mice after 8 weeks of dietary intervention (*n* = 6). **(E)** Distance to one-time forcefulness in each group of mice after 8 weeks of dietary intervention (*n* = 6). **(F,G)** Forelimb maximum grip strength and relative grip strength of mice in each group after 8 weeks of dietary intervention (*n* = 6). Values are given as mean ± SEM. a, significantly different from NOC; b, significantly different from BI; c, significantly different from KI; d, significantly different from OC.

A total of 110 male C57BL/6 J mice aged 6 to 8 weeks were obtained from Skbex Biotechnology, Anyang, Henan. These mice were housed in a specific pathogen-free (SPF) laboratory at Zhengzhou University. The mice were kept in cages with *ad libitum* access to food and water, under controlled conditions of an ambient temperature of 22 ± 2°C, relative humidity of 47–55%, and a light–dark cycle of 12 h each. The mice were divided into four groups (Figure 1A). Following a week of acclimatization, 18 randomly selected mice served as the non-obese control group (NOC) and were fed a balanced diet (AIN-93G, henceforth “ND”). The remaining 92 mice were fed a high-fat diet (D12492, “HFD”) with a fat content of 60% for a duration of 8 weeks to induce obesity. Obesity was defined as a body weight that was more than 20% greater than the average body weight of the non-obese control group. The obese mice were then randomized divided into three groups: the balanced diet intervention group (BI, *n* = 18), the ketogenic diet (D10070801, “KD”) intervention group (KI, *n* = 18), and the obesity control group (OC, *n* = 18), which continued to be fed the high-fat diet. The composition of the diets is presented in Table 1. Food consumption was measured daily and body weight was recorded weekly throughout the study. The experimental procedures for animal care and use were conducted in accordance with the guidelines set by the National Institutes of Health (NIH). The protocols for these animal experiments were approved by the Zhengzhou University Life Sciences Institutional Review Board (Approval number: ZZUIRB 2021–146).

**Table 1:** Diet contents.

	*ND* (AIN-93G)		*KD* (XTKD01)		*HFD* (D12492)	
Protein (gm%)	20		17		26.2	
Carbohydrate (gm%)	64		0		26.3	
Fat (gm%)	7		67		34.9	
Protein (kcal%)	20.3		10		20	
Carbohydrate (kcal%)	63.9		0		20	
Fat (kcal%)	15.8		90		60	
Total kcal/gm	3.9		6.7		5.24	
Ingredient	gm	kcal	gm	kcal	gm	kcal
Casein, 30 Mesh	200	800	100	400	200	800
L-Cystine	3	12	1.5	6	3	12
Corn Starch	397	1,590	0	0	0	0
Maltodextrin	132	528	0	0	125	500
Sucrose	100	400	0	0	68.8	275.2
Cellulose	50	0	50	0	50	0
Soybean Oil	70	630	0	0	25	225
Lard	0	0	0	0	245	2,205
Cocoa Butter	0	0	406	3,654	0	0
Total	952	3,960	557.5	4,060	716.8	4017.2

### 2.2 Strength and exercise test

After 8 weeks of implementing a dietary intervention, a total of 24 mice (six from each group) were selected randomly for exercise performance tests. The mice underwent three tests to measure their maximal grip strength, using a grip strength meter from Bioseb in France. The resulting data were analyzed by calculating the average maximal grip strength. Following the grip strength tests, the mice participated in a one-time exhaustion test on a treadmill. Prior to the formal test, the mice underwent adaptive training and exercised on the treadmill for 10 min at a speed of 10 m/min for three consecutive days. During the exhaustion test, the treadmill slope was set to 0. The mice were subjected to three speed gradients: 10 m/min for 15 min, 15 m/min for 15 min, and 20 m/min until exhaustion (23). The duration of exhaustion was recorded. Any mice that were unable to continue moving forward after 10 s of continuous electrical stimulation were deemed exhausted. The electrical stimulation used was set to 1 Hz with an intensity of 0.3 mA. Immediately following exhaustion, all 24 mice were anesthetized and euthanized. Samples were taken from them to test for oxidative stress and antioxidant indices.

### 2.3 Preparation of serum and tissue samples

The mice were fasted for 12 h and allowed free access to water. They were then anesthetized by injection of sodium pentobarbital. Blood was taken from the abdominal aorta and left at room temperature for 1 h. After that, it was centrifuged at 5,000 g for 10 min. The serum was collected and promptly transferred to the −80°C refrigerator for preservation. The epididymis white fat, liver, and gastrocnemius muscle of the mice were swiftly extracted, frozen in liquid nitrogen and transferred to −80°C refrigerator for storage.

### 2.4 Indirect calorimetry

Indirect calorimetry was performed on mice using the Columbus CLAMS-12 system (Columbus, OH, United States). The system enables simultaneous and real-time monitoring of multiple parameters, including oxygen consumption, carbon dioxide production, voluntary activity, heat expenditure and respiratory exchange rate (RER). A total of 24 mice (*n* = 6) were introduced to the CLAMS-12 system for 1 day to acclimatize to the environment, and then monitored the next whole day with data recorded.

### 2.5 Histological analysis

Fresh white adipose tissue was fixed in a fixative for ga period exceeding 24 h. The tissue underwent various dehydration gradients before being immersed in white fat dip wax and embedding. Paraffin slices were then trimmed and sectioned using a paraffin slicer. These slices were subsequently stained with hematoxylin and eosin (H&E staining), dehydrated and sealed with neutral gum. Finally, the slices were observed under an inverted microscope (Olympus, Japan), the resulting images were collected and analyzed. Six regions were randomly selected under a 40 magnification field of view, and the cell diameter was measured and counted using ImageJ software.

### 2.6 Serum biochemical analysis

The serum levels of TC (total cholesterol), TG (triglycerides), HDL-C (high-density lipoprotein), LDL-C (low-density lipoprotein), ALT/AST (alanine aminotransferase/aspartate aminotransferase), CK (creatine kinase), LDH (layered double hydroxides), and BUN (blood urea nitrogen) were measured using reagent kits (Nanjing Jiancheng Bioengineering Institute, Nanjing, China). The instructions provided with the kits were followed meticulously to ensure accurate results.

### 2.7 Liver and muscle antioxidant capacity index

To prepare the tissue samples, begin by cutting approximately 80 mg of tissue into an EP tube. Next, add an appropriate proportion of saline to the tube, based on the amount of tissue. Once the saline has been added, use a grinder to homogenize the tissue. The recommended homogenization time is 99 s, at a frequency of 60 Hz, and should be repeated 2 to 3 times. After homogenization, centrifuge the homogenate at 3000 r/min for 15 min. Carefully remove the supernatant from the tube, as this will be the sample used for testing. Reagent kits (Nanjing Jiancheng, Nanjing, China) are utilized to measure the levels of SOD, GSH-PX, T-AOC, CAT, and MDA in the sample. Following the instructions provided with the kits, perform the necessary assays to measure each of these antioxidants and oxidative stress markers.

### 2.8 Quantitative real-time PCR

Total RNA was extracted from liver and gastrocnemius tissue using the Total RNA Isolation Kit (Beibei Biotech, Zhengzhou, China). The extracted total RNA was subjected to reverse transcription using the HiScript III RT SuperMix kits with gDNA wiper (Vazyme Biotech, Nanjing, China). Real-time quantification of gene expression was performed using the UltraSYBR Mixture (Applied Biosystems, CWBIO) on a PCR amplification system (Roche LightCycler 480II). The relative gene expression of 18 s was calculated using 2^−ΔΔ*C*^T method. The primer sequences involved in the experiment are shown in Table 2.

**Table 2:** Primer sequences of target genes.

Genes	Primer sequence(F)	Primer sequence(R)
*Sod1*	GGAGGTGGTGATAGCCGGTAT	TGGGTAATCCATAGAGCCCAG
*Gpx1*	CTTGCAGAAGAAATACGCCAT	CTTCTTTCCCGATAATATCTGT
*Cat*	CGCAAGGCCATCGACTACATCC	CGTCTCCACCACTTCGGGTT
*Nrf2*	TACAACAATGAGCCTGCGAAC	CATCAAATGAGGGCAATCCG
*18 s*	GTAACCCGTTGAACCCCATT	CCATCCAATCGGTAGTAGCG

18 s: 18 s ribosomal RNA; Sod1: Superoxide dismutase 1; Gpx1: Glutathione peroxidase 1; Nrf2: Nuclear factor erythroid2-related factor 2; Cat: Catalase.

### 2.9 Statistical analysis

The data were analyzed using SPSS 26.0 and graphing was performed using GraphPad Prism 8.0. One-way ANOVA followed by Tukey’s test was used to compare the data between the two groups, while a T-test was used to compare the data within each group. The data were presented as mean ± SEM, and *p* < 0.05 was set as statistically significant.

## 3 Results

### 3.1 Weight change and energy intake

After 8 weeks of dietary intervention, the prevalence of obesity in mice reached 58%, with a total of 30 mice exhibiting obesity. The BI (balanced diet intervention) and KI (ketogenic diet intervention) groups demonstrated a gradual decrease in weight throughout the intervention, which was significantly lower than the OC (obesity control) group (*p* < 0.001) and not statistically different from the NOC (normal control) group at week 8 (Figure 1B). These findings suggest that a KD (ketogenic diet) and ND (normal diet) can effectively reduce body weight in obese mice, while mice fed a HFD (high-fat diet) continue to rapidly gain weight into adulthood. Regarding energy intake, the BI group had the lowest energy intake, followed by the NOC group, which was significantly lower than the other three groups (*p* < 0.001). The OC group exhibited the highest energy intake, followed by the KI group, which was significantly higher than both the NOC and BI groups (*p* < 0.001; Figure 1C).

### 3.2 Ki enhances exercise endurance performance in mice

Due to its association with changes in muscle mass, increased oxidative stress, and inflammation, obesity often affects exercise performance. To investigate this relationship, we conducted exhaustion tests and maximum grip measurements in mice. Figures 1D,E shows that the one-time exhaustion time (*p* < 0.05) and exhaustion distance (*p* < 0.01) were significantly shorter in the OC group compared with the NOC group. In contrast, after 8 weeks of ketogenic dietary intervention, mice in the KI group exhibited significantly higher running time and distance compared to the OC group (*p* < 0.01). There was no significant difference in running performance between the KI, NOC, and BI groups. Interestingly, the obese mice treated with BI also experienced improved endurance, although not significantly different from the OC group. In addition, there were no significant differences in maximum grip strength among the four groups of mice (Figure 1F). However, the relative gripping strength of the NOC group was significantly higher than that of the other three groups (*p* < 0.01; Figure 1G). These findings suggest that KI improves exercise endurance in HFD-induced obese mice but does not have a significant effect on muscle explosiveness.

### 3.3 Ki increases energy expenditure and fat oxidation

**Figure 2:**
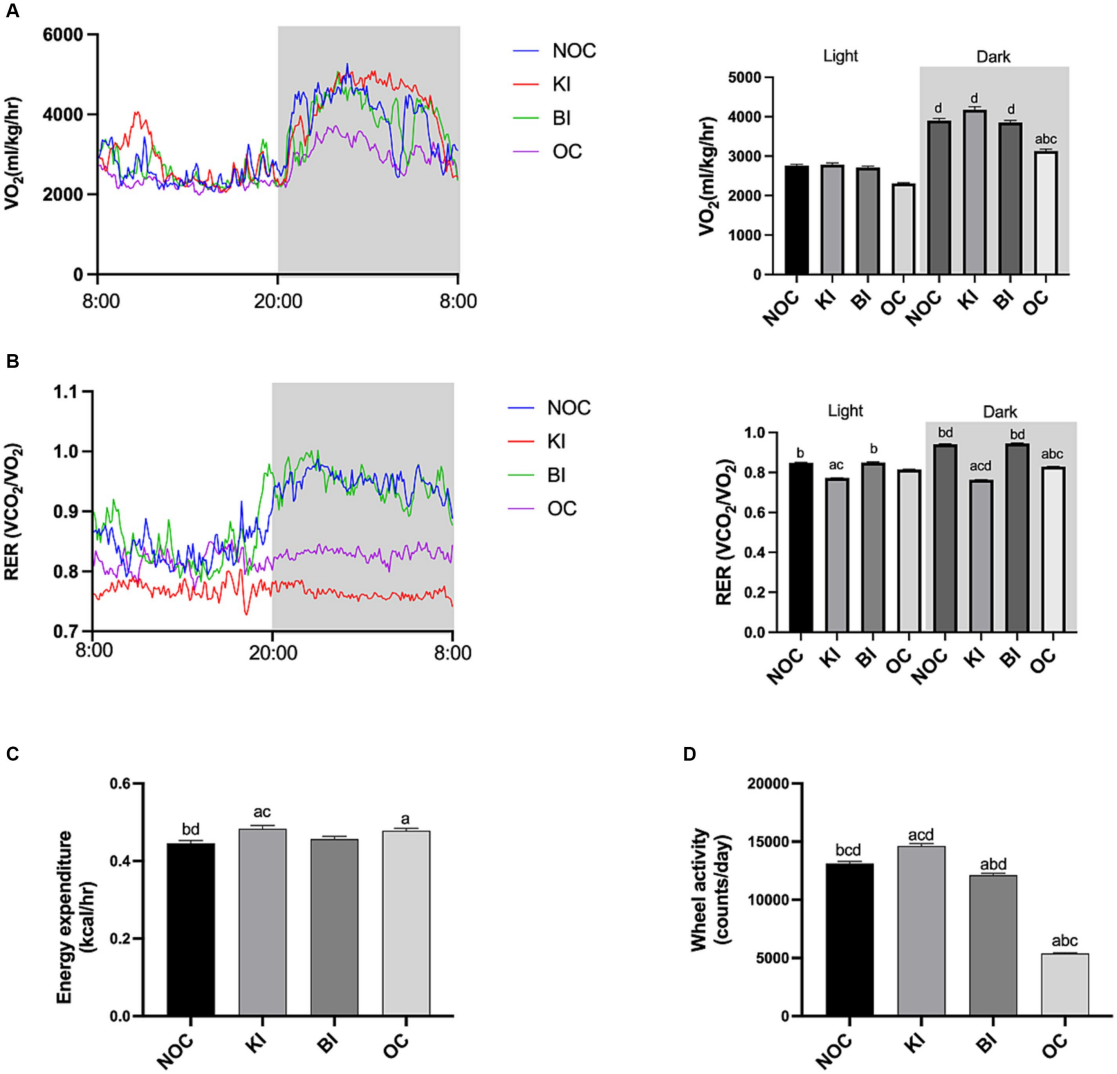
Energy metabolism in mice. **(A)** O2 consumption of each group mice. **(B)** RER (VCO2/VO2) of mice. **(C)** Energy expenditure. **(D)** Voluntary activity. *n* = 6 for each group. Values are given as mean ± SEM. a, significantly different from NOC; b, significantly different from BI; c, significantly different from KI; d, significantly different from OC.

Obesity is commonly associated with an imbalance in energy expenditure and energy intake. We used CLAMS-12 system to monitor energy metabolism in mice. As shown in Figure 2A, the dark oxygen consumption of mice in OC group was significantly lower than that in the other three groups (*p* < 0.01). Notably, the KI group of mice had the highest dark oxygen consumption. The data of RER showed that KI group was lower than that of the other three groups, especially in the dark (*p* < 0.0001; Figure 2B). The RER value of the KI group mice approached 0.7, indicating that KI group mice mainly used fat as the substrate for energy supply. However, NOC and BI groups were more likely to use carbohydrates as the energy substrate for energy supply, and the RER level of the BI group recovered to the level of NOC group. Interestingly, RER levels in OC group were significantly lower than those in NOC and BI groups. This indicates that with the increase of fat content in the diet, the substrate of energy consumption will be transferred to fat. In addition, the average energy expenditure of the KI group was significantly higher than that of the BI and NOC groups (*p* < 0.05; Figure 2C). Unsurprisingly, obesity limited the voluntary wheel activity in the OC group, while the KI group was significantly higher activity compared to the other three groups (*p* < 0.01; Figure 2D). In summary, our findings demonstrate that the 8-week ketogenic diet improved basal energy metabolism in obese mice.

### 3.4 Fat loss over diet intervention

**Figure 3:**
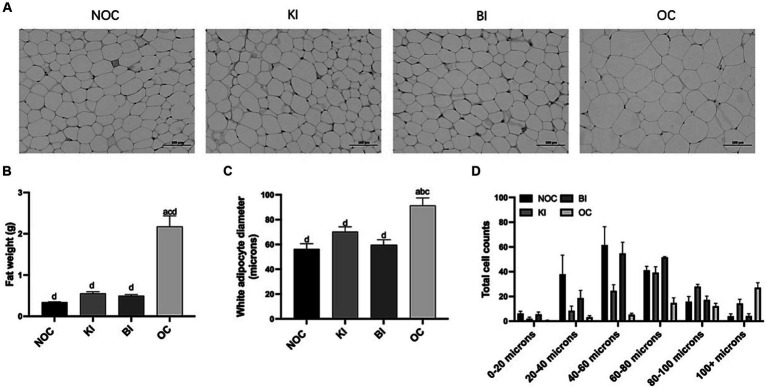
Effect of dietary intervention on morphology of white fat. **(A)** Representative HE staining of fat sections (Scale bars = 100 μm, *n* = 6). **(B)** The weight of fat (*n* = 6). **(C)** Average white adipocyte diameter (*n* = 5). **(D)** White adipocyte diameter range classification (*n* = 6). Values are given as mean ± SEM. a, significantly different from NOC; b, significantly different from BI; c, significantly different from KI; d, significantly different from OC.

We collected epididymal white fat (henceforth fat) from mice to evaluate the weight loss effect of dietary intervention. Compared to the OC group, the fat weight in the KI and BI groups decreased by 75 and 77%, respectively. Conversely, the OC group demonstrated an 84% increase in fat content compared to the NOC group (Figure 3B). Furthermore, the fat diameter in the KI and BI groups was significantly lower than in the OC group (*p* < 0.01), and not significantly different from the NOC group. Notably, the adipocyte diameter in the BI group closely resembled to that of the NOC group, as opposed to the KI group (Figures 3A,C,D). These results indicate that KI and BI can ameliorate the problems of fat accumulation and fat droplet enlargement caused by HFD.

### 3.5 Changes of blood lipids under dietary intervention

**Figure 4:**
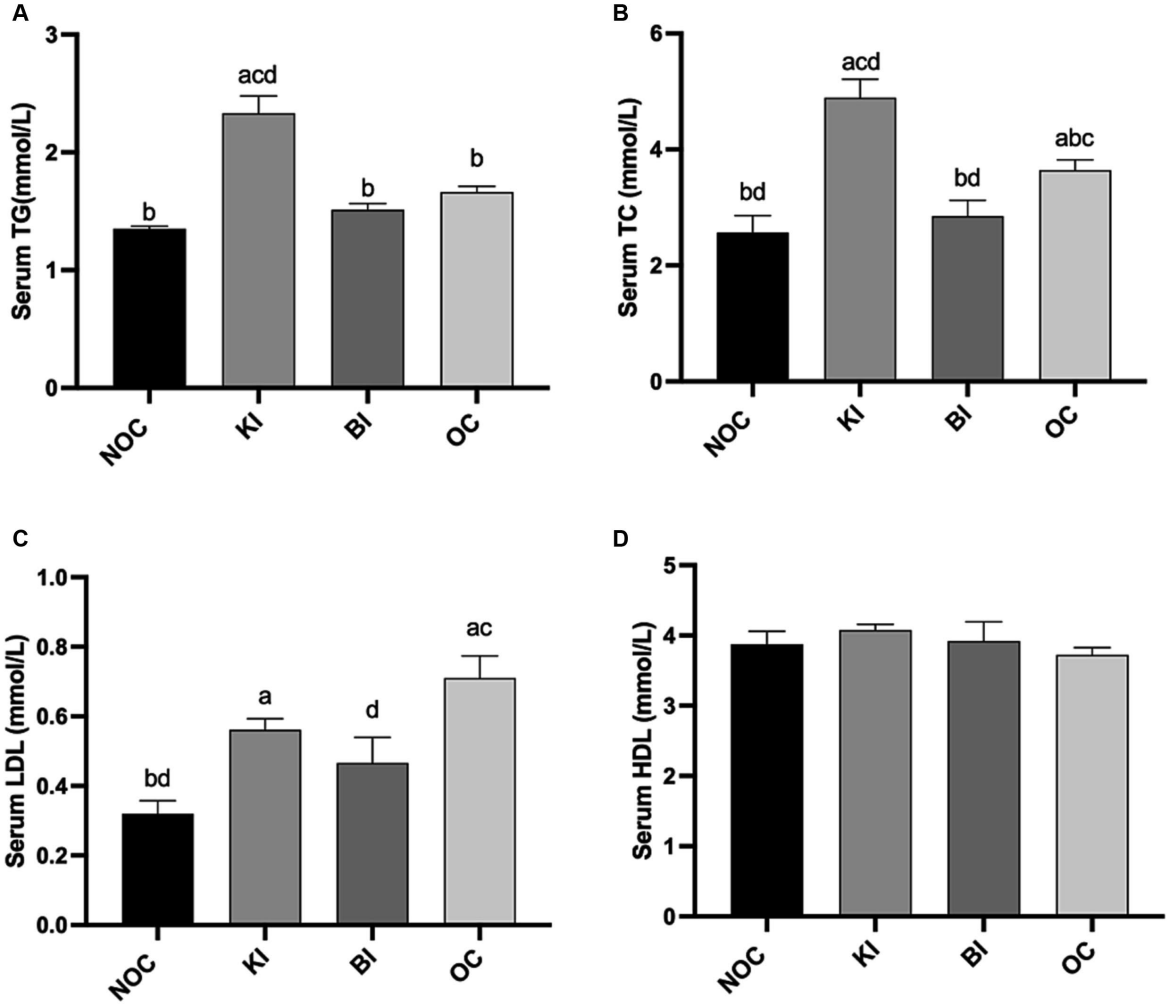
Effect of dietary intervention on serum **(A)** TG, **(B)** TC, **(C)** LDL, **(D)** HDL. *n* = 6 for each group. Values are given as mean ± SEM. a, significantly different from NOC; b, significantly different from BI; c, significantly different from KI; d, significantly different from OC.

As depicted in Figures 4A,B, the administration of KI resulted in a marked increase in serum TC and TG levels, which were significantly higher than those in the OC group (*p* < 0.05). Conversely, the BI group displayed significantly lower serum TC levels compared to the OC group, with no significant difference observed between the BI group and the NOC group (*p* < 0.05). In addition, LDL level were significantly reduced in the BI group compared to the OC group (*p* < 0.05), surpassing the efficacy of the KI group (Figure 4C). HDL levels were not significantly different between the 4 groups (Figure 4D). These findings suggest that KI may contribute to the development of potential lipid disorders. However, BI ameliorated dyslipidemia in HFD-induced obese mice.

### 3.6 Changes of oxidative stress damage indexes

**Figure 5:**
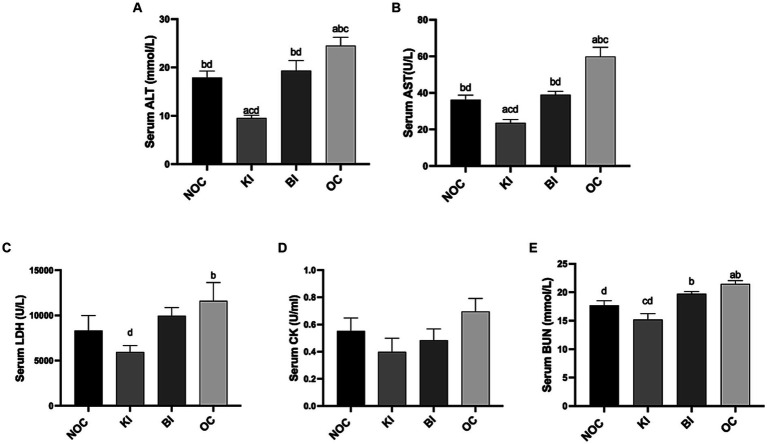
Serum oxidative stress and damage index levels. Effect of dietary intervention on serum **(A)** ALT, **(B)** AST, **(C)** LDH, **(D)** CK, **(E)** BUN. *n* = 6 for each group. Values are given as mean ± SEM. a, significantly different from NOC; b, significantly different from BI; c, significantly different from KI; d, significantly different from OC.

The measurements of ALT and AST are crucial in assessing liver damage. In Figures 5A,B, it was demonstrated that KI effectively lowers serum ALT and AST levels, while also mitigating liver injury induced by a HFD in comparison to the OC group (*p* < 0.01). Similarly, BI significantly reduced liver injury markers in obese mice, while also restoring them to levels similar to the NOC group (*p* < 0.05). In addition, KI significantly reduced serum LDH compared with OC group (*p* < 0.05; Figure 5C). Although there was no significant difference in serum CK levels among the 4 groups, KI and BI showed a decreasing trend compared with the OC group (Figure 5D), indicating that KI and BI could reduce the risk of liver and skeletal muscle diseases caused by high fat diet to a certain extent. BUN, as an indicator for evaluating renal function, decreased significantly in the KI group compared to the BI and OC groups (*p* < 0.01; Figure 5E).

### 3.7 Effects of dietary intervention on antioxidant capacity of liver and muscle

**Figure 6:**
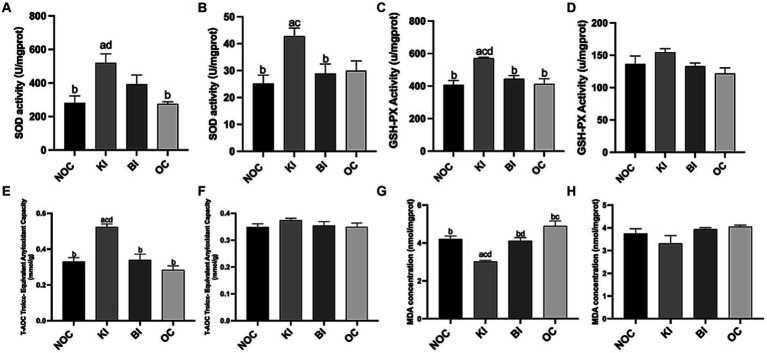
Antioxidant index **(A,B)** SOD, **(C,D)** GSH-PX, **(E,F)** T-AOC, **(G,H)** MDA activity in liver and gastrocnemius. *n* = 6 for each group. Values are given as mean ± SEM. a, significantly different from NOC; b, significantly different from BI; c, significantly different from KI; d, significantly different from OC.

**Figure 7:**
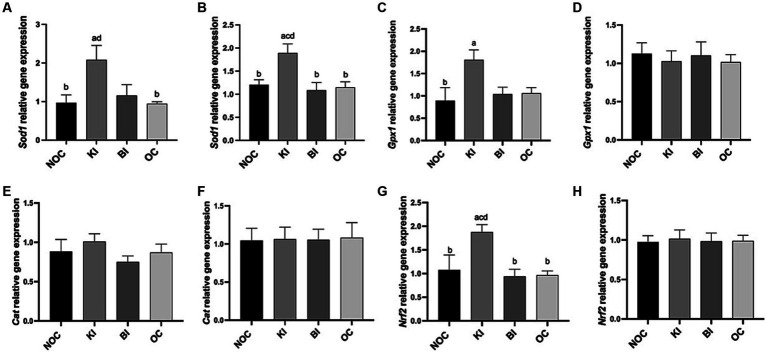
Relative expression of antioxidant genes **(A,B)** *Sod1*, **(C,D)** *GPX1,* **(E,F)** *Cat*, **(G,H)** *Nrf2* in liver and gastrocnemius. *n* = 6 for each group. Values are given as mean ± SEM. a, significantly different from NOC; b, significantly different from BI; c, significantly different from KI; d, significantly different from OC.

As shown in Figure 6A, liver SOD activity was significantly higher in the KI group than in the NOC and OC groups (*p* < 0.01), while in gastrocnemius, SOD activity was significantly higher in the KI group than in the NOC (*p* < 0.01) and BI (*p* < 0.05) groups (Figure 6B). Consistent with these findings, the mRNA expression of *Sod1* in both the liver and gastrocnemius of the KI group showed a similar trend (Figures 7A,B). In addition to the increase in SOD activity, the KI group also exhibited a significant increase in the activity of the antioxidant index GSH-PX enzyme in the liver of mice (Figure 6C), and the liver *Gpx1* gene expression of KI group also increased compared with the other three groups (Figure 7C), but there was no significant difference in gastrocnemius among the four groups (Figure 7D). The activity of hepatic antioxidant enzyme T-AOC was significantly higher in the liver of the KI group compared to the remaining three groups (Figure 6E). Furthermore, the level of MDA, an indicator of membrane lipid peroxidation, was significantly lower in the liver tissue of the KI group compared to the NOC, BI and OC groups (Figure 6G). But in gastrocnemius, there were no significant differences in antioxidant indexes GSH-PX, T-AOC and MDA levels among the four groups (Figures 6D,F,H). Interestingly, there were no significant differences observed in the expression of the antioxidant gene *Cat* in both the liver and gastrocnemius among the 4 dietary intervention groups (Figures 7E,F). However, as Nrf2 serves as a key transcription factor regulating antioxidant stress and plays an important role in inducing the body’s antioxidant response, it is noteworthy that its gene expression was significantly up-regulated in the liver of the KI group (Figure 7G).

## 4 Discussion

Obesity is widely acknowledged as a significant public health hazard. The primary health risks of obesity are mediated through metabolic disorders induced by excess fat, potentially resulting in type 2 diabetes, cardiovascular disease, and premature (24–26) mortality. Moreover, there is a notable correlation between obesity and skeletal muscle (24, 27) commonly manifesting as hip or knee osteoarthritis, and inducing a systemic inflammatory response. This is a leading, persistent source of pain, disability, and diminished quality of life (28). The ketogenic diet is a well-established method for weight management, enhancing exercise endurance by preserving glucose and gluconeogenesis reserves through ketone metabolism, which mimics the effects of starvation (29). It is worth investigating the impact of the ketogenic diet on exercise ability of individuals with obesity after weight loss interventions, as well as examining its effects on oxidative stress levels and antioxidant capacity. While rodents are commonly utilized in preclinical studies focusing on these issues, a limitation of previous research is the lack of obese mouse models. In this study, we addressed this limitation by utilizing an obese mouse model system. Our study showed that an 8-week ketogenic diet intervention significantly improved the aerobic exercise capacity and enhanced antioxidant capacity in the liver of obese mice. However, it is important to note that this intervention also posed potential risks, such as elevated blood lipid concentrations.

Previous studies have shown that both the ketogenic (KI) and balanced diets (BI) have weight loss effects in both obese humans and mice (30, 31), which is consistent with the findings of this study. Our data showed that after 8 weeks of dietary intervention, the body weight of the KI group was lower than that of the BI group, although there was no significant difference in weight loss between the two groups. Although the caloric intake of the KI group was the same as that of the OC group, the body weights of the mice in the KI group were significantly lower, which may be due to the increase in energy expenditure and the elevated metabolic rate of fat oxidation. In the study of Yamazaki et al., a 3-week ketogenic diet significantly reduced body weight in obese mice, and the weight loss was greater than the normal diet intervention (32), while in another human study, BI was more effective at weight loss than KI (33). However, studies by Ma et al. showed that 8-week KI mice had lower body weight than BI mice (20). Differences in findings may be related to feed ingredient ratios, palatability, and duration of intervention, but there is no denying that both the ketogenic diet and the balanced diet can play a role in weight loss. In our study, the KI group had higher body fat and fat adipocyte size, despite significantly reducing body weight in obese mice, suggesting that we still need to be cautious about its safety.

The impact of a ketogenic diet and exogenous ketone supplements on exercise performance is a hot topic in the sports industry (29, 34). Our study examined the effects of an 8-week ketogenic diet intervention on obese mice. The results showed that the ketogenic diet significantly improved the one-time exhaustion time and exhaustion distance of obese mice. However, it had no significant effect on the maximum grip strength of obese mice. The liver produces ketone bodies through metabolism and releases them into the bloodstream with the assistance of a ketogenic diet. The benefits of ketone body metabolism include the preservation of glucose and gluconeogenesis reserves, enabling a source of oxidizable carbon for energy supply of the brain (29). During mitochondrial oxidative phosphorylation, ketone body oxidation leads to the conservation of free energy in ATP, providing a thermodynamic advantage over other carbon substrates (35) and making ketone bodies the most energy-efficient fuel (34). Ma et al. showed that 8-week KI improves exercise performance by upregulating ketolysis and fatty acid oxidation and prevents muscle damage by modulating IL-6 secretion (36). Huang et al. pointed out that KI intervention for 8 weeks significantly improved the exhaustive exercise ability of normal mice (12). In addition, we noticed that the exercise endurance of mice in the BI group was also somewhat improved compared to the OC group after 8 weeks of dietary intervention but the effect was weaker than that of the KI group. This may be due to weight loss following a balanced diet that lessens the limitations on exercise capacity caused by obesity. Excess fat puts mechanical stress on the joints, and obesity with or without metabolic dysfunction may limit exercise capacity to some extent (37). Interestingly, the maximal and relative grip strengths of obese mice did not change significantly after dietary intervention, whereas the relative grip strengths of mice in the NOC group were significantly higher than those of the remaining three groups, which may be attributed to the degradation and disruption of muscle function caused by obesity, whereas the KI and BI interventions did not improve the muscle explosive strength significantly. It has been reported that KI may counteract the effects of resistance training and lead to muscle loss (38). Another study showed that the ketogenic diet had no effect on athletes’ anaerobic exercise performance (39). However, it has also been shown that a ketogenic diet significantly improves anaerobic performance in resistance-training men (40). The different results may be related to the testing method, dietary rationing and combination of exercise, and further research is still needed to prove this. The role of branched-chain amino acids (BCAA) in myasthenia gravis and injury protection has been widely reported, and some studies suggest that BCAA supplementation combined with a ketogenic diet may be preferable for maintaining muscle mass (41–43).

The respiratory exchange rate (RER), which is the ratio of VCO2/VO2, is used to measure the respiratory quotient (RQ) during steady state. Remarkably, the RER differed significantly among the four groups of mice in this study, which were subjected to different dietary interventions (*ND*, *KD*, and *HFD*). Interestingly, the RER curves for the NOC and BI groups, which received the same dietary interventions, were nearly identical. However, the KI group, despite achieving similar weight loss results, exhibited a significantly lower RER value (approaching 0.7). This indicates that the KI group primarily relied on fat as a substrate, while the BI and NOC groups primarily relied on carbohydrates for energy. These findings suggest that BI and KI promote weight loss through distinct mechanisms: the BI group achieved weight loss by significantly reducing energy intake compared to the KI and OC groups, whereas the KI group regulated energy substrate utilization to control body weight. Ketosis has the potential to alter the competition for respiratory substrates and enhance oxidative energy metabolism, particularly during endurance exercise. Therefore, incorporating a ketogenic diet may unlock greater metabolic efficiency in the human body (29).

High serum TC and TG are closely associated with many health problems including atherosclerosis, type 2 diabetes and coronary heart disease (44, 45). Lei et al. found that a high-fat diet for 11 weeks exhibited significantly higher TC and TG levels compared to non-obese mice on a normal diet (46). Similarly, another study showed that a 4-week high-fat diet significantly increased TC and TG levels in diabetic rats (47). However, Gu et al. showed that an 8-week KI reduced serum TC and TG levels in obese patients (48). This seems to go against the intuition that high dietary fat causes dyslipidemia, but dietary fat is not the only driver of serum TC and TG levels. Other studies have reported that ketogenic diets lower serum TC and TG levels due to less liver fat accumulation (49, 50). In our study, 8-week KI significantly increased serum TC and TG levels in obese mice, which was consistent with our previous 4-week intervention results (51). Interestingly, it has been reported that serum TC and TG are reduced with increasing duration of KI in mice (52). In our study, both KI and BI reduced body weight in obese mice, but KI significantly elevated serum TC and TG levels relative to NOC and OC, whereas the BI group did not. This suggests that hyperlipidemic symptoms induced by high dietary fat may not be reduced by weight loss, but may be further worsened by KI.

The effectiveness of the ketogenic diet in preventing organ damage has also been reported, and BUN and ALT are often used as markers of exercise-related acute kidney injury and acute liver injury. In previous studies, both markers were significantly reduced by KI immediately and 24 h after exhaustive exercise (10, 12), which is consistent with our experimental results. Blood levels of CK and LDH are usually used as muscle-damage markers. After intense exercise, plasma CK of KI intervention mice decreased significantly, while plasma CK of BI mice increased significantly in a previous study (36). MDA is a kind of lipid peroxide, and the excessive accumulation of MDA in the body is one of the main causes of energy supply injury. The study of McAllister et al. showed that low-carb high-fat diet significantly reduced the blood MDA level of athletes (53). In the present study, liver tissue MDA was significantly lower in the KI group than in the BI, NOC, and OC groups, whereas there was no significant difference in muscle, suggesting that the KI reduced liver cell peroxidation. Oxidative stress is commonly defined as a state of over-presentation due to impaired reactive oxygen species (ROS) overproduction and/or elimination (54). The antioxidant and oxidative stress mitigating effects of ketone bodies *in vitro* and *in vivo* have been widely described, especially in neuroprotection. Nagao et al. showed that ketone body metabolism can be reprogrammed to maintain different cellular signaling, redox potentials, or metabolic demands (55). It has been reported that a high-fat diet fed to ketogenic-deficient mice resulted in extensive hepatocellular damage and inflammation (54). Other conflicting reports suggest that high ketone body concentrations induce oxidative stress. High doses of β-Hydroxybutyrate (βHB) or AcAc induced decreased nitric oxide secretion, lipid peroxidation, and SOD, GSH-PX, and CAT in calf hepatocytes (56, 57). This difference may result from differences in ketone production caused by different dietary ratios, with physical ketosis occurring in response to changes in the ratios of the three macronutrients in the ketogenic diet. Even if an increase in protein from 5% kcal to 10% kcal significantly reduces ketosis, a tiny protein calorie restriction may have complex metabolic implications (54). Ketogenic diets and low-carbohydrate diets are often considered an unpleasant eating style that may lead to dyslipidemia, liver steatosis, constipation, hyperuricemia, and nephrolithiasis (51, 58, 59), which makes adhering to a ketogenic diet for the long term challenging. The long-term effect of ketogenic diet on mice is still unclear, and ketogenic diet has certain potential and limitations. Previous studies have shown that ketogenic diet combined with aerobic exercise and nutritional supplements is safer and more effective (43, 59). In addition, supplementing the intake of fruits, vegetables, and grains may provide additional benefits such as micronutrients that were not considered in our study.

There are several limitations in our study that need to be addressed. Firstly, we only used male mice to avoid interference caused by the estrus cycle of female mice. However, this approach overlooks the potential differences in results between genders, and further research on this aspect is necessary. Secondly, the extrapolation of our results to humans is constrained by the isogenic background of the mice used in our study. Therefore, further experimental validation is required to establish the relevance of our results to human subjects. In addition, cocoa butter has been reported to contain flavonoids, anthocyanins, caffeine, catechins and epicatechin, which have antioxidant activity and are desirable properties for the prevention of reactive oxygen species (ROS) (60–62). Among them, flavonoids and polyphenols have been shown in numerous studies to enhance aerobic performance (63–65). These may interfere with the benefits of the ketogenic diet *per se*. Lastly, the specific molecular mechanisms underlying the ketogenic diet’s ability to enhance exercise endurance and provide antioxidant properties still require investigation.

## 5 Conclusion

In the present study, we established an obese mouse model through a high-fat diet, and evaluated the effects of *KD* and *ND* on body weight, exercise performance, energy metabolism, oxidative stress level and antioxidant capacity of obese mice from the perspective of obesity-related metabolic homeostasis. Our results suggest that the ketogenic diet can effectively improve the performance of treadmill endurance in obese mice after weight loss and enhance the liver antioxidant capacity. But we also reveals the safety risks and limitations of the extreme proportion of ketogenic diet. The combination of the ketogenic diet and other supplements needs to be further studied to maximize the improvement of the ketogenic diet strategy by using the ketogenic diet as a cyclical dietary intervention.

## Data availability statement

The original contributions presented in the study are included in the article/supplementary material, further inquiries can be directed to the corresponding author.

## Ethics statement

Ethical approval was not required for the study involving humans in accordance with the local legislation and institutional requirements. Written informed consent to participate in this study was not required from the participants or the participants’ legal guardians/next of kin in accordance with the national legislation and the institutional requirements. The animal study was approved by Life Sciences Institutional Review Board of Zhengzhou University (Approval number: ZZUIRB 2021–146). The study was conducted in accordance with the local legislation and institutional requirements.

## Author contributions

YW: Conceptualization, Data curation, Software, Visualization, Writing – original draft, Writing – review & editing. YD: Data curation, Formal analysis, Software, Visualization, Writing – original draft, Writing – review & editing. YZ: Investigation, Writing – original draft. JY: Methodology, Writing – review & editing. CR: Methodology, Writing – review & editing. HM: Data curation, Writing – review & editing. ZC: Conceptualization, Project administration, Supervision, Writing – review & editing.
